# Photocatalytic disinfection of surfaces with copper doped Ti0_2_ nanotube coatings illuminated by ceiling mounted fluorescent light

**DOI:** 10.1371/journal.pone.0197308

**Published:** 2018-05-16

**Authors:** Tilen Koklic, Štefan Pintarič, Irena Zdovc, Majda Golob, Polona Umek, Alma Mehle, Martin Dobeic, Janez Štrancar

**Affiliations:** 1 Jožef Stefan Institute, Ljubljana, Slovenia; 2 NAMASTE Center of Excellence, Ljubljana, Slovenia; 3 Veterinary faculty, University of Ljubljana, Ljubljana, Slovenia; Louisiana State University Health Sciences Center, UNITED STATES

## Abstract

High economic burden is associated with foodborne illnesses. Different disinfection methods are therefore employed in food processing industry; such as use of ultraviolet light or usage of surfaces with copper-containing alloys. However, all the disinfection methods currently in use have some shortcomings. In this work we show that copper doped TiO_2_ nanotubes deposited on existing surfaces and illuminated with ceiling mounted fluorescent lights can retard the growth of *Listeria Innocua* by 80% in seven hours of exposure to the fluorescent lights at different places in a food processing plant or in the laboratory conditions with daily reinocuation and washing. The disinfection properties of the surfaces seem to depend mainly on the temperature difference of the surface and the dew point, where for the maximum effectiveness the difference should be about 3 degrees celsius. The TiO_2_ nanotubes have a potential to be employed for an economical and continuous disinfection of surfaces.

## Introduction

Economic burden of $30–80 billion was estimated by the Center for Disease Control and Prevention (CDC) for the annual number of foodborne illnesses, affecting 48 million Americans [1,2]. Over 320.000 cases of food-borne zoonotic diseases were evidenced in humans each year, thus the measures in view of food safety have to be very strict especially on food and food premises hygiene [3]. Food can become contaminated at any point during production and distribution, as well as in consumers’ own kitchens. Therefore, foodborne illness risk reduction and control interventions must be implemented at every step throughout the food preparation process [4]. Recent global developments are increasingly challenging international health security according to the World Health Organization (WHO). These developments include the growing industrialization and trade of food production and the emergence of new or antibiotic-resistant pathogens. Micro-organisms are known to survive on surfaces, for extended periods of time. Among the foodborne pathogens, *Listeria monocytogenes* has the highest mortality rate in humans and is one of the most environmentally resistant facultative anaerobic bacteria growing at its optimal temperatures from -18 °C to 10 °C, in environments with or without oxygen with propensity of forming a biofilm [5]. Between 13 serotypes of *Listeria monocytogenes*, three serotypes (1/2a, 1/2b and 4b) are the reason for the majority of human listeriosis [6]. In our previous research using pulsed-field gel electrophoresis typing of *L*. *monocytogenes* isolates from poultry abattoir we identified the same serotype (classical 1/2a, molecular IIa) with the exception of one isolate with a different serotype (4b, IVb), mainly found on the surface, but some also in the air [7].

Many disinfectants were tested in the prevention of *Listeria monocytogenes* contamination, however organic burdening and biofilm formation effectively inhibited disinfectants’ microbicidal activity [8,9]. Although biofilm formation is common for every environment where microorganisms are close to the surface, its formation is even more problematic in the food industry, where remains of foods in inaccessible places enable survival and the multiplication of *Listeria*. It was speculated that specific properties of persistence of *L*. *monocytogenes*, might be the reason for spreading of persistent strains of *L*. *monocytogenes* across the surfaces of food-processing plants, but also by transferring meat products between different plants [10,11]. In addition, some studies report about the possibility of reduced *L*. *monocytogenes* susceptibility to some chemical disinfectants [12]. Permanent maintenance of hygiene in food processing industry is therefore of utmost importance for the continuous reduction in the number of bacteria. For this reason, regular cleaning and disinfection is mandatory, but it is often performed poorly and irregularly specially when parts of the meat processing equipment are inaccessible [13]. Namely the risk for food contamination arise mainly due to low hygiene of food premises and not from previously contaminated animals as it was shown by Ojeniyi et al. [14], and by our own work, where we were unable to confirm the transfer of *L monocytogenes* from broiler farm to the abattoir, since we couldn’t prove a positive case of *L monocytogenes* on broiler farms among the investigated animals [7]. One of the main reasons for spreading of the persistent strains of *L*. *monocytogenes* might be its ability of enhanced adherence to surfaces in a relatively short time [15,16], therefore the continuous antibacterial function of food contact surfaces should be implemented. One of such continuous disinfection methods, suitable for disinfection of the air, liquids and surfaces is the use of ultraviolet light (UV), which is being employed as one of the physical methods of decontamination in the food processing industry [17]. Short-wave ultraviolet light (UVC, 254 nm) was shown to be effective against wide spectrum of bacteria, viruses, protozoa, fungi, yeasts and algae, by altering cell DNA [17]. However, UVC has limited applicability in food industry since it can cause sunburn, skin cancer, and eye damage under direct exposure. UVC lights can also produce ozone, which can be harmful to human health, and finally materials exposed to UVC light for longer period age faster, especially plastics and rubber, which break down under UVC exposure. On the other hand, long-wave ultraviolet light (UVA, >320 nm) as a part of a sunlight, not absorbed by the atmosphere ozone layer, thus reaching the earth’s ground, and is not harmful to human health, can still cause some oxidative damage, however has much weaker effect on microorganisms than UVC [17]. Since UVA is normally present as a small part of the fluorescent lighting spectrum, one could use ceiling mounted fluorescent lights for permanent surface disinfection provided that the oxidative damage of UVA light at a surface could be enhanced. This can be achieved by illuminating TiO_2_ deposited on a surface by UVA light. Namely, illuminated TiO_2_ is known to produce reactive oxygen species, such as hydroxyl or superoxide radicals, which can also be used for disinfection of surfaces. As early as in 1977 it has been shown that TiO_2_ can decompose cyanide in water when illuminated with sunlight [18]. If TiO_2_ is irradiated with photons with energies greater than material’s band gap (E_g_) electron-hole pairs are generated—for TiO_2_ with E_g_ around 3 eV wavelengths below approximately 415 nm are needed [19]. Photo generated holes are highly oxidizing whereas photo generated electrons are reducing enough to produce superoxide from dioxygen [20]. After reacting with water, holes can produce hydroxyl radicals (·OH). Photo excited electrons can become trapped and loose some of their reducing power, but are still capable of reducing dioxygen to superoxide radical (·O_2_^-^), or to hydrogen peroxide H_2_O_2_. Hydroxyl radical, superoxide radical, hydrogen peroxide, and molecular oxygen could all play important roles in preventing proliferation of bacteria. Using TiO_2_ surface coatings one should therefore be able to maintain clean surfaces with the use of UV light close to visible spectrum.

Doping of TiO_2_ with transition metals such as copper has already been sugested to enhance its photocatalytic properties [21,22]. For example, several research groups made antibacterial coatings based on TiO_2_ containing copper and tested them at different light intensities [23–29]. Although the antibacterial effect of copper doped TiO_2_ was greater compared to TiO_2_ alone, much of the antibacterial effect is due to leached copper ions [25,26]. It turned out that higher amounts of copper than approximately 1% cannot be successfully incorporated into TiO_2_ structure, but are deposited as clusters on the TiO_2_ surface [30–32], from which copper can be released into the solution. Although this contributes to toxicity of the nanoparticles in absence of irradiation [33] it also leads to unacceptable development of copper resistant bacterial strains as well. Our goal was therefore to incorporate small amount of copper into TiO_2_ structure, thus avoiding formation large copper aggregates, which might leach into the solution.

We have shown previously that Cu^2+^-doped TiO_2_ nanotubes (Cu-TiO_2_NTs) coated polymer surfaces reduce number of seeded bacteria by 99.93% (i.e. Log_10_ reduction = 3.2, when inoculated with 2.08 10^4^ *Listeria innocua*) when illuminated with low power UVA diodes for 24 hours at 4 °C [34], where the intensity of UVA light needed to observe the antibacterial effect was only about 10 times more than it is usually present in common fluorescent lighting. In this manuscript we present antibacterial effect observed on polymer surfaces when illuminated with common fluorescent lights, which emit light with strong peak at 365 nm and were already present on a ceiling of a food processing plant. Coated surfaces innoculated with 10^7^ bacteria exhibit similar antimicrobial effect as we observed previously on the TiO_2_ nanotube coated petri dishes, reducing the number of *Listeria innocua* by 80% in seven hours of exposure to the fluorescent lights, compared to control surfaces without the nanotube coating.

## Materials and methods

### Materials

The spin trap, 5-(Diethoxyphosphoryl)-5-methyl-1-pyrroline-N-oxide (DEPMPO) (Alexis, Lausen) was used as purchased without further purification and stored at -80 °C. The spin-trap stock solutions were always freshly prepared. Ethanol (EtOH) and methanol (MeOH) from Merck AG (Darmstadt, Germany) were used in Lichrosolv^®^ gradient grade quality. Media and culture materials were obtained from Gibco—Invitrogen Corporation (Carlsbad, California).

### Preparation of bacterial inoculum

Antimicrobial properties were tested on non-pathogenic bacterium *Listeria innocua*, which is closely related to pathogenic species *Listeria monocytogenes*. Suspension of *Listeria innocua* strain, isolated during routine examination (RDK.), was supplied by the Institute of Microbiology and Parasitology, Veterinary faculty, University of Ljubljana. Strain was maintained frozen at -70 °C in sterile vials containing porous beads which serve as carriers to support microorganisms (Microbank, pro-lab Diagnostics) and kept at -70 °C. The inoculum was prepared in liquid medium and incubated aerobically for 24 h at 37 °C. After incubation the culture contain approximately 10^9^ colony forming units (CFU) per milliliter. Working suspensions with appropriate concentrations were achieved by several 10-fold dilutions.

### Preparation and properties of Cu^2+^-doped TiO_2_ nanotubes

Cu^2+^-doped TiO_2_ nanotubes (Cu-TiO_2_NTs) were prepared in several steps: (i) first sodium titanate nanotubes (NaTiNTs) were synthesized from anatase powder (325 mesh, ≥ 99.9%, Aldrich) and 10 M NaOH (aq) (Aldrich) at T = 135 °C for 3 days under hydrothermal conditions. Exact synthesis procedure is described previously [35], (ii) in the next step NaTiNTs were rinsed with 0.1 M HCl(aq) yielding protonated titanate nanotubes (HTiNTs), (iii) then 400 mg HTiNTs were dispersed in 100 mL of 0.5 mM solution of Cu^2+^(aq) (source of the Cu^2+^ was CuSO_4_·5H_2_O (Riedel de Haen)) using an ultrasonic bath (30 minutes) and stirred at room temperature for 3 hours. By centrifugation the solid material was separated from the solution, and (iv) finally isolated material was heated in air at 375 °C for 10 hours.

The powder X-ray diffraction (XRD) pattern was obtained on a Bruker AXS D4 Endeavor diffractometer using Cu Kα radiation (1.5406 Å; in the 2*θ* range from 10 to 65°). Morphology of the particles in the sample was determined using transmission electron microscope (TEM, Jeol 2100). The specimen for the TEM investigation was prepared by dispersing the sample in MeOH with the help of an ultrasonic bath and depositing a droplet of the dispersion on a lacey carbon-coated copper grid.

### Activity of TiO_2_ nanotubes

The photocatalytic activity of synthesized titanate and TiO_2_ nanomaterials was determined using electron paramagnetic resonance spectroscopy (EPR) with spin trapping, which was optimized for measurement of primary radicals generated in the vicinity of the nanomaterial surface. This was achieved by measuring primary hidroxyl radicals in the presence of 30% ethanol with 5-(diethoxyphosphoryl)-5-methyl-1-pyrroline-N-oxide spin trap (DEPMPO). EPR spin trapping was applied to measure the generation of reactive oxygen species (ROS) production.

### Deposition of Cu-TiO_2_NTs on PET surface and testing of the deposition stability

The deposition of Cu-TiO_2_NTs was made on different surfaces: 2.5 cm × 7.5 cm polyethylene terephthalate (PET) slides, polystyrene petri dishes (8 cm diameter), aluminum oxide slides with the same dimensions as PET slides. The surfaces were washed before deposition. They were soaked in 20% NaOH solution, rinsed with distilled water, and finally with ethanol vapor.

The suspension of the nanotubes with concentration of 1 mg/mL was processed with ultrasonic liquid processor (Sonicator 4000, Misonix) prior to the deposition on the surfaces. Sonication was performed using 419 Microtip^™^ probe, 15 min process time, 10 s pulse-ON time, 10 s pulse-OFF time and maximum amplitude (resulting in 52 W of power).

The surfaces were treated with compressed air 3 times for 3 s. 150 μL of nanoparticle suspension was applied on each surface, immediately after compressed air treatment, and smeared evenly. The same number of surfaces with nanoparticle deposition and control surfaces were prepared for each experiment. On control surfaces, only 150 μL of solution was applied. After the deposition, the surfaces were left in the oven at 50 °C for 2 h. Then they were rinsed with distilled water and put back in the oven at 50 °C for another 2 h.

The photocatalytic activity of the nanodeposit on the surfaces was tested using EPR spectroscopy. Three measurements were performed on the surfaces, with or without the nanodeposit. On each surface, small pool, proportionate to the size of the sample, was made with silicon paste and was filled with 2 μL of 0,5 M DEPMPO and 18 μl of 30% ethanol and irradiated with 290 nm diode for 5 min. The diode was 1–2 mm above the surface of the sample. The solution with short-lived radicals being trapped in the form of stable DEPMPO spin adducts was then drawn into the quartz capillary of 1 mm diameter, which was put in the 5 mm wide quartz tube and transferred into EPR spectrometer. All EPR measurements were performed on an X-band EPR spectrometer Bruker ELEXYS, Type W3002180. All measurements were recorded at room temperature using 1 Gauss (10^−4^ T) modulation amplitude, 100 kHz modulation frequency, 20 ms time constant, 15 x 20 seconds sweep time, 20 mW microwave power and 150 G sweep width with center field positioned at 3320 G.

The amount of deposited material was estimated from EPR signal decrease when rinsing the deposit of 150 μL of 1 mg/mL applied to a 2.5 × 7.5 = 18.8 cm^2^ surface. With EPR signal being decreased to about 1/3, we estimated that the amount of deposited nanomaterial was about 2 μg/cm^2^.

### Antimicrobial activity of nanotube coated PET surface in a meat processing plant

Four measurement points were selected in a poultry slaughterhouse with regards to different air microclimate conditions (humidity, temperature, airflow) as well as intensity of UV irradiation. PET slides were innoculated with 10^7^ bacteria in 10 μL droplet and placed either vertically or horizontally at different altitudes (0.5 or 2 m) and exposed for 7 hours. After exposure, the samples were washed in saline (NaCl 0.9%) and examined bacteriologically to determine the number of bacteria. Survival of bacterial culture of *Listeria innocua* has been measured for samples with and without germicidal Cu-TiO_2_NTs coating. Reduction ratio was expressed in percentage and logarithm (Log_10_). % reduction was calculated using the following equation:

$$\%\text{R}=(1-{\text{N}}_{\text{final}\;\text{control}}/{\text{N}}_{\text{final}\;\text{Cu}-\text{TiO}}{}_{{}_{2}\text{NTs}})*100,$$ (1)

Where N_final control_ is the number of bacteria after the exposure on a control surface, and N_final Cu-TiO_2_NTs_ is the number of bacteria after the exposure on a surface coated with Cu-TiO_2_NTs nanotubes.

Log reduction was calculated using the following equation:

$$LR=-\text{lo}{\text{g}}_{10}({\text{N}}_{\text{final}\;\text{Cu}-\text{Ti}O_{2}}{}_{\text{NTs}}/{\text{N}}_{\text{final}\;\text{control}})=\text{lo}{\text{g}}_{10}\left({\text{N}}_{\text{final}\;\text{control}}\right)-\;\text{lo}{\text{g}}_{10}({\text{N}}_{\text{final}\;\text{Cu}-\text{Ti}{\text{O}}_{2}}{}_{\text{NTs}})$$ (2)

### Antimicrobial activity in presence of repeated daily contamination and washing

Effect of long term illumination was studied only on PET surfaces, by placing uncoated (control) PET slides and PET slides covered by Cu-TiO_2_NTs on cooled (4 °C) aluminum plates in order to mimic cold and condensing conditions at the cooling walls commonly present in food processing plants. Bacterial suspension (10 μl) of living microorganism *Listeria innocua* in concentration of 1.5 to 5.0 x 10^9^ CFU/mL was applied daily on each PET slide. The slides were then cooled to the dew point, which prevented the drying of microorganism containing droplets on the slides. Slides were washed with 100 mL of sterile saline solution (0.9 weigth % NaCl) at different time intervals and the number of surviving microorganisms was determined. The remaining PET slides were stored in the dark at 4 °C until the next day when the above described process was repeated. The whole experiment with daily washing and bacteria application lasted for 28 days.

## Results and discussion

### Structure and photochemical activity of copper doped TiO_2_ nanotubes (Cu-TiO_2_NTs)

**Fig 1:**
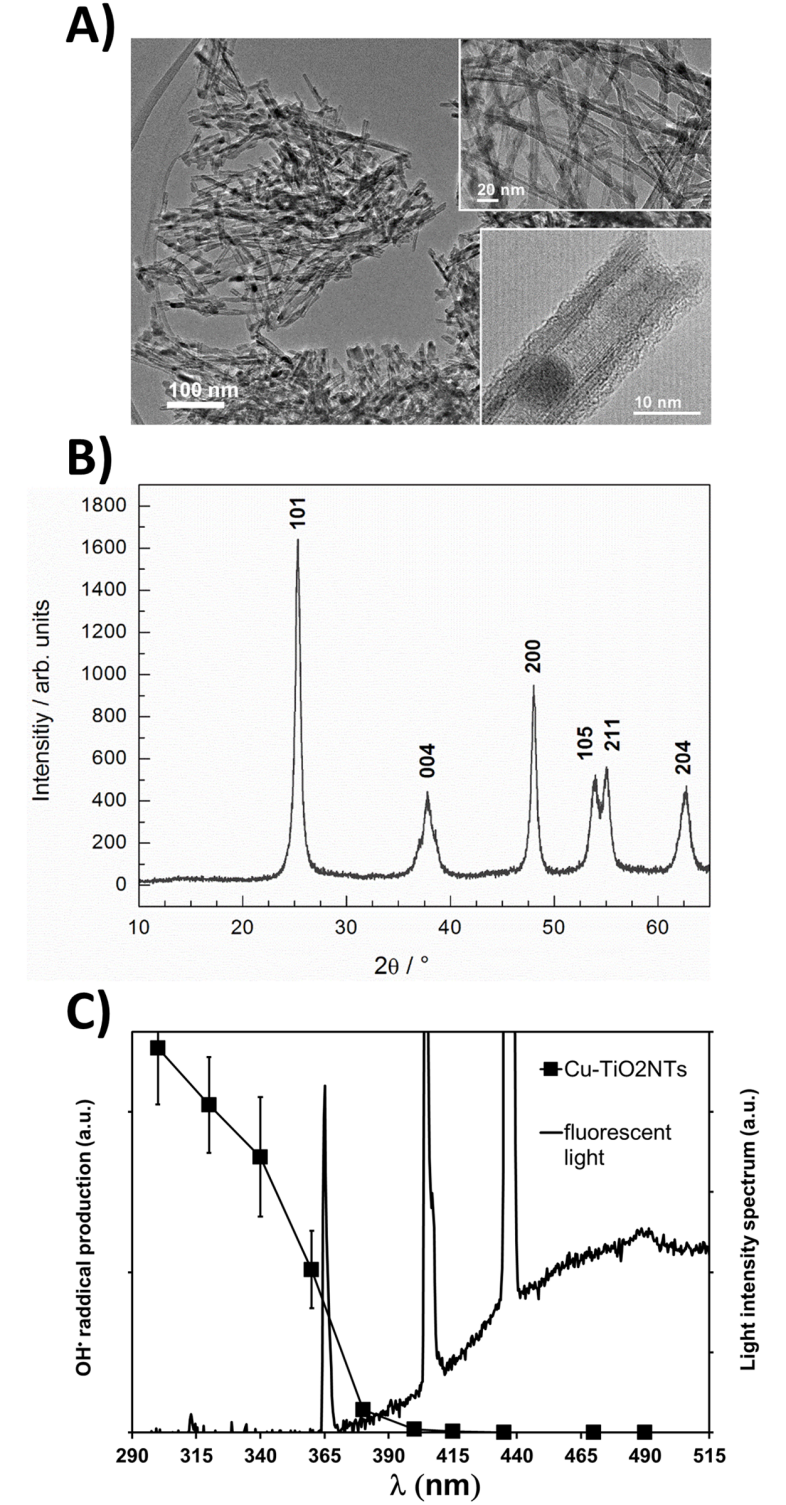
Structure and photocatalytic activity of Cu^2+^-doped TiO_2_ nanotubes (Cu-TiO_2_NTs) excited at different wavelengths. A) Transmission electron microscopy (TEM) images of the nanotubes taken at different magnifications. The nanotube wall structure can be clearly seen from a lower high resolution (HRTEM) image shown in the inset; B) XRD of the nanotubes. All the diffraction peaks in the diffractogram are indexed and correspond to anatase; C) Amount of hydroxyl radical production (closed squares) versus illumination of the nanotubes at different wavelengths is shown against emitted light spectrum of a common fluorescent light (black line).

Cu^2+^-doped TiO_2_ nanotubes used in this study were prepared by conversion of H_2_Ti_3_O_7_ nanotubes doped with Cu^2+^ by calcination at 375 °C. H_2_Ti_3_O_7_ nanotubes upon calcination above 350 °C transform to anatase nanotubes [36]. As it can be observed from transmission electron microscopy (TEM) image (Fig 1A) during this transformation the nanotube morphology is preserved while nanotubes become shorter [37]. Porous structure of Cu^2+^-doped TiO_2_ nanotubes is clearly seen from both insets to Fig 1A. The outer diameter of nanotubes varies from 8 to 10 nm while in length they reach up to 300 nm. The phase identification of the sample was performed using X-ray powder diffraction (XRD). All diffraction peaks (Fig 1B) are indexed to anatase TiO_2_ (ICCD card no. 89–4203) and no diffraction peaks for CuO are observed, indicating that the copper content is below 3 wt. % and/or also that CuO particles are very small. Results of elemental analysis, performed with scanning electron microscopy (SEM), showed that copper content is about 1.5 wt. % (content in at. %: Ti-32.9, O-66.5 and Cu-0.6; EDS spectrum is shown in the supplement S3 Fig). With more detailed TEM investigation at higher magnifications (lower inset to Fig 1A) nanoparticles caught in the inner side or outer sided of the tubes were observed but the number of this particles is low due to the low copper doping. When copper content is higher, that is about 11 wt. %, CuO nanoparticles are clearly observed as it was shown in our previous study[31]. More detailed characterization, using advanced high resolutiom transmission electron microscopy techniques, of the copper doped TiO_2_ nanotubes is reported in Koklic et al. [34] (article submitted).

We have previously shown that the Cu-TiO_2_NTs deposited on polystyrene petri dishes reduce up to 99.93% (3.2 log_10_ reduction, initial number of bacteria 2.08·10^4^) *Listeria innocua* in 24 hours in a refrigerator at 100% humidity, illuminated with UVA light emitting diodes [34]. However, Usage of additional illumination results in additional costs associated with application of such disinfection methods. On the other hand, ceiling mounted fluorescent lights, which are already in use in many food processing plants, contain a small portion of emitted light in UVA range. Fig 1C shows the spectrum of emitted light by a ceiling mounted fluorescent lamp. Three peaks in the spectrum are clearly visible, with one spectral peak at 365 nm. It is this peak which is absorbed by the Cu-TiO_2_NTs, as it is evident from the absorption of light as a function wavelength (Fig 1C, closed squares). The absorption of light versus wavelength of the light is consistent with a bandgap of the TiO_2_, a property of a semiconductor such as TiO_2_. Matsunaga et al. showed already in 1985 that Escherichia coli cells were completely sterilized when TiO_2_ was irradiated with high intensity UV light.[38] Since then the antibacterial effect of photoexcited TiO_2_ was shown against a wide range of microorganisms.[27] Photocatalytic mechanism and related photochemistry of TiO_2_ is well researched[24,39–41,41–45], antibacterial action seems to depend mainly on ·OH radicals, which are produced on the surface of TiO_2_ when illuminated with light consisting of wavelengths below TiO_2_’s bandgap. Due to this semiconductor property of the Cu-TiO_2_NTs nanotubes the production of hydroxyl (·OH) radicals on the surface of nanotubes increases with decreasing wavelength. We measured the amount of produced radicals as a function of different wavelengths of light, by using a DEPMPO spin trap (Fig 1C, closed squares), which is commonly used for efficient trapping of the hydroxyl radicals [46]. Since the spectrum of the emitted light from a ceiling mounted common fluorescent light (Fig 1C, black line, the peak at 365 nm) overlaps with the spectrum of the light needed for efficient photoexcitation of the nanotubes (the closed squares), we expected that the nanotube coated surfaces could be excited by fluorescent lights, which are already present on ceilings at food processing plants. Especially due to intense peak at 365 nm, which is present in the emitted spectrum of the fluorescent light bulb and represents about 1% of total light emitted by the lamp. The scale in Fig 1C is set to emphasize the fluorescent light emission peak at 365 nm, which is overlapping with the ·OH radical production efficiency curve (Fig 1C closed squares) and seems to be responsible for the antimicrobial activity of the nanotubes.

### Deposition stability of copper doped TiO_2_ nanotubes on different surfaces

**Fig 2:**
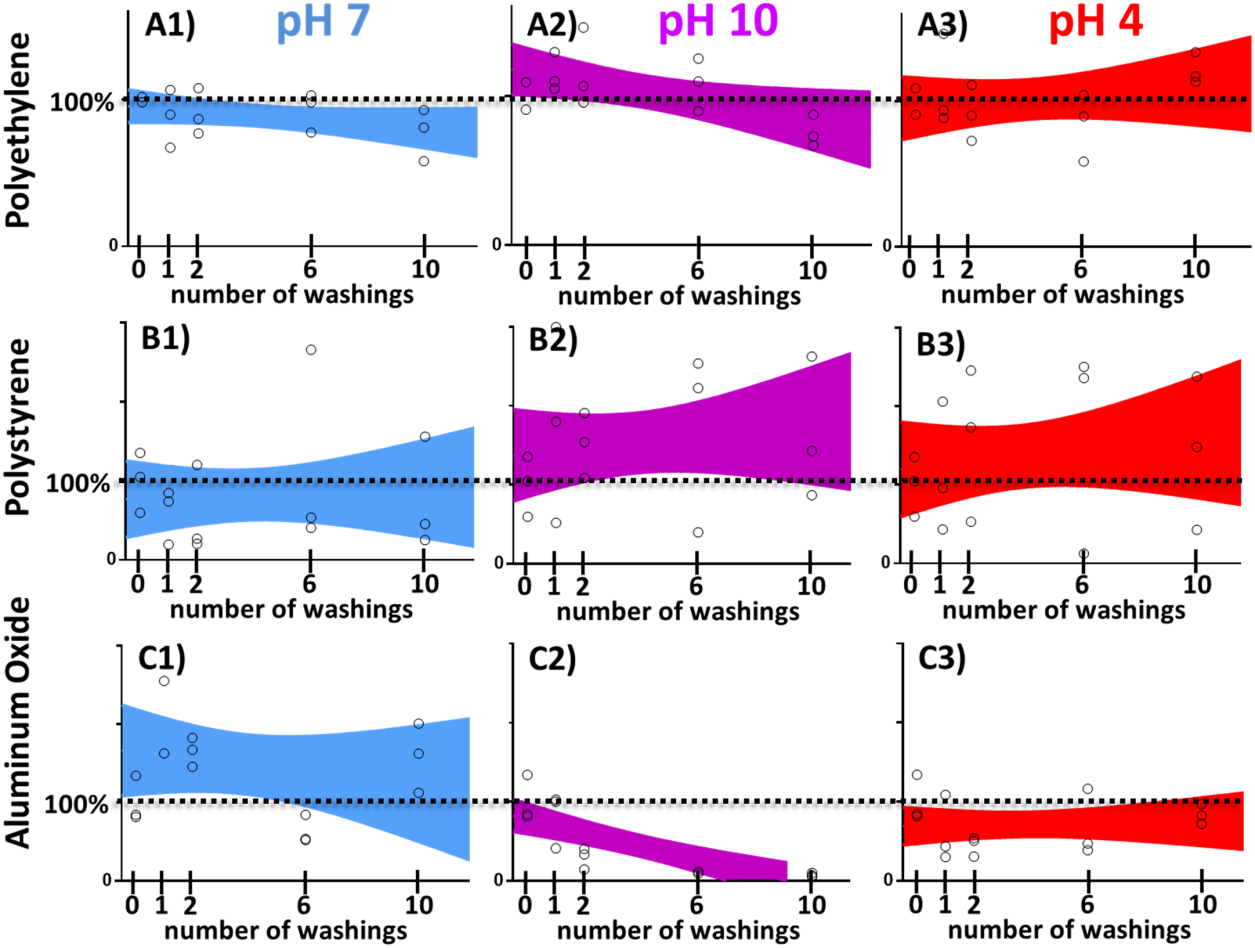
Deposition stability of copper doped TiO_2_ nanotube coatings on different surfaces. Stability on the surface against washing at different pH conditions (neutral, pH7—blue, acidic, pH4 –red, basic, pH10 –violet), without abrasion, but under extensive water flow. Individual measurements are shown as open circles; colored areas represent 90% confidence areas, which enclose the area that one can be 90% sure to contain the linear fit curve.

Next we tested the stability of the nanotubes deposited on different surfaces, which are commonly used in food processing industry. The dispersion of Cu-TiO_2_NTs was added to the clean surface (see Materials and methods) and left to dry. No special chemical modification of either nanotubes or surface was necessary. Unattached nanoparticles were washed away under a stream of water and the production of hydroxyl radicals was measured as described in Materials and Methods section. Since the amount of the produced radicals is proportional to the amount of Cu-TiO_2_NTs still remaining on the surface after extensive washing, we used the measurement of the the quantity of radicals produced by illuinated surfaces as a measure for the stability of the deposition. That is, if the amount of produced radicals remains constant throughout the washing cycles, then the Cu-TiO_2_NTs nanoparticles should remain attached to the surface. We tested different materials: polyethylene terephtalate (Fig 2, row A), polystirene (Fig 2, row B), and aluminum oxide (Fig 2, row C). All surfaces were repeatedly soaked at different pH conditions neutral (pH7, Fig 2, first column, blue color), basic (pH10, Fig 2, second column, violet color), and acidic (pH4, Fig 2, third column, red color) and extensively washed under a stream of water after each soaking. The amount of material versus washing step was fit with a linear curve using GraphPad Prism version 7.00 for Windows (GraphPad Software, La Jolla California USA, www.graphpad.com). The area, which contains a linear fit, which describes the data with 90% certainty is shown on each graph. In all of the graphs, except for aluminum oxide washed at pH10, linear fit is contained around 100% deposited material (horizontal dotted lines), thus indicating that Cu-TiO_2_NTs nanoparticles deposited to various surfaces should withstand daily washings with different detergents commonly used in food processing industry. However the material will not provide long term disinfection of aluminum oxide surfaces, when washed with basic detergent (Fig 2, frame C2). The Fig 2, frame C2 shows that deposited nanotubes can be washed off the aluminum oxide surface when washed in alkaline conditions. Recently Berg et al.[47] showed that both TiO_2_ and aluminum oxide nanoparticle surfaces have high negative surface charge at pH 10, which might result in a repulsive force between the two surfaces.

### Antimicrobial activity of TiO_2_ nanotube coated surfaces placed in a food processing plant

**Fig 3:**
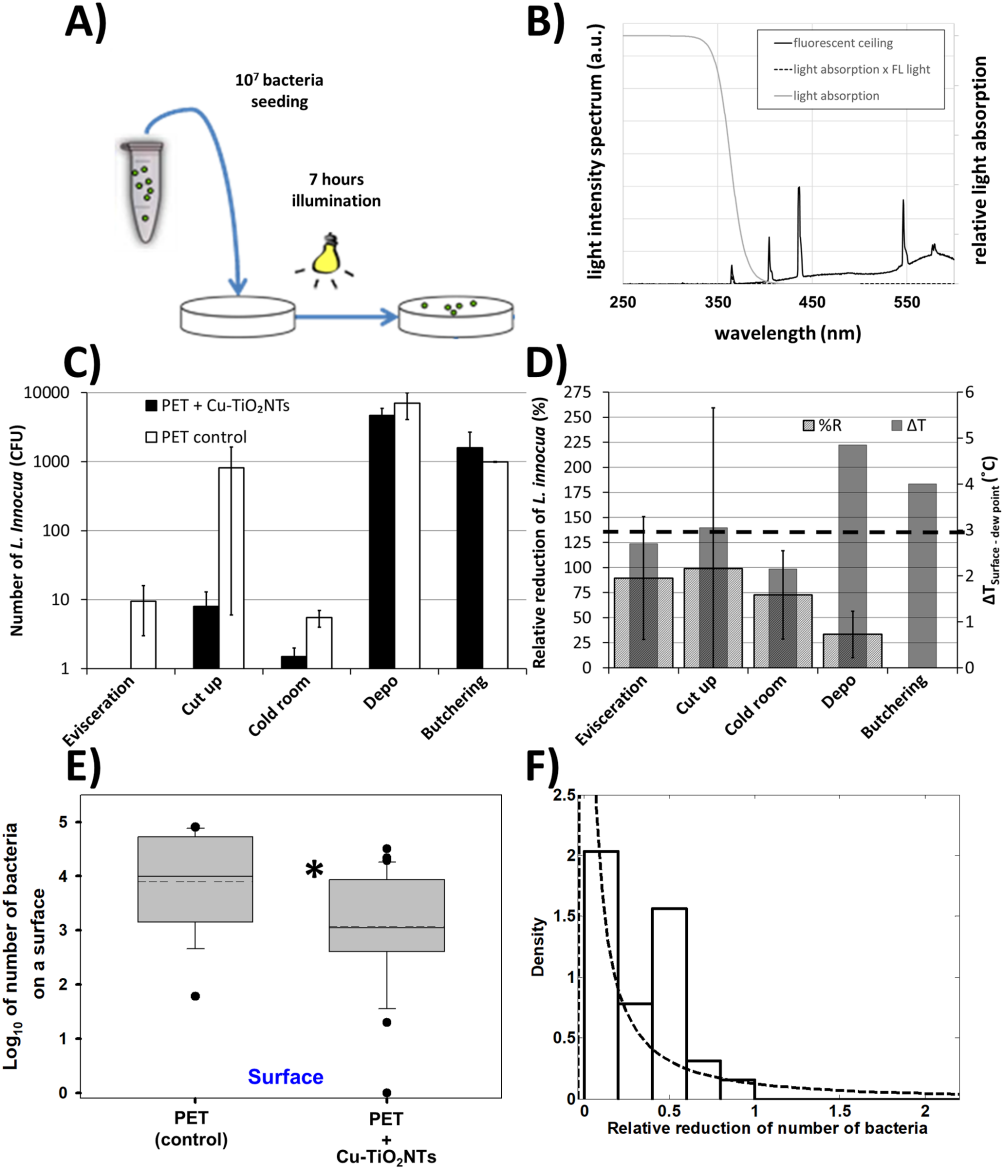
Survival of Listeria innocua under exposure to ceiling mounted fluorescent lights in a food processing plant. **A)** Schematic presentation of the experiment. **B)** spectrum of emited light from ceiling mounted fluorescent lights (black line); relative light absorption by the Cu-TiO_2_NTs (grey line); spectrum of absorbed light by the nanotubes (dashed line). **C)** Number of Listeria innocua shown on a logarithmic scale. On each surface 10^7^ bacteria were placed and left at different places in the food processing plant for 7 hours. After this time period remaining bacteria were transferred from the surfaces and colony forming units (CFU) were counted. Number of CFU on control surfaces is shown with white bars (PET control); Number of CFU on the nanoparticle coated surfaces is shown with black bars (PET + Cu-TiO_2_NTs); **D)** Relative reduction (%R) of bacteria as a consequnce of disinfecting action of nanoparticle coated surface, illuminated with ceiling mounted fluorescent lights (wide textured bars), calculated according to the Eq 1 in Materials and Methods section; Grey bars represent the microclimatic parameter (difference between surface temperature and dew point temperature—dew point spread) which correlates best with the %R. **E)** Boxplot presents log10 of number of bacteria on either control PET surface without antibacterial nano coating (PET) or the number of bacteria on a PET surface coated with copper doped TiO_2_ nanotubes (PET+Cu-TiO_2_NTs). Median value of each distribution is shown with horizontal line within each box, while the dashed line marks the mean. The boundary of the box closest to zero indicates 25th percentile, and the boundary farthest from zero indicates the 75th percentile. Whiskers (error bars) above and below the box indicate the 90th and 10th percentiles. The outliers are shown as dots. Note the LOG scale. Since normality test (Shapiro-Wilk) failed (P < 0.050), Mann-Whitney rank sum test was performed on the control group (N = 16, median = 4) and on the PET+Cu-TiO2NTs group (N = 32, median = 3.1). The difference in the median values between the two groups is greater than would be expected by chance (P = 0.003)*. **F)** Histogram of the distribution (probability density function—PDF) of survival ratios, calculated as a ratio between the number of Listeria innocua from the surface with antibacterial coating (PET+Cu-TiO_2_NTs) and the number of Listeria innocua from a control surface without the coating (PET). For inefficient antibacterial coating survival ratio of 1 is expected. Dotted line is lognormal fit of the distribution with a maximum of the PDF below 0.1 for the reduction of the number of bacteria on the surface.

We exposed polyethylene terephthalate (PET) surfaces with or without Cu-TiO_2_NTs antibacterial coating at different locations in the food processing plant to test whether the intensity of ceiling mounted fluorescent lights in a food processing plant is sufficient to provide measurable antibacterial activity of surfaces coated with the nanotubes. We applied 10 μL of bacterial suspension of *Listeria innocua* on the PET surfaces (10^7^ bacteria), as shown schematically in Fig 3A, and placed the PET surfaces at different places in the food processing plant with respect to performed tasks (evisceration, meat cut up, cold room, Depo—meat storage, and butchering) for 7 hours. The plant used common ceiling mounted fluorescent lights with a light emitting spectrum shown in Fig 3B. The air microclimate conditions (UV light Intensity, humidity, and temperature) were followed and the number of remaining bacteria was determined (S1 and S2 Tables). Microclimatic air conditions measured at different places in the food processing plant were different with respect to temperature, humidity, ambient light intensity emitted from ceiling mounted fluorescent lights, and airflow (S1 Fig). The reduction of the number of bacteria compared to a control surface was achieved at two places: 1) Evisceration (90%±61%), and 2) Cold room (73%±44%) (Fig 3D, grey bars). As seen in S1 Table log_10_(CFU) = 0 and cannot be shown on the logarithmic scale of the Fig 3C. Since the error in calculating %R is very large due to errors in determining colony forming units (Fig 3C) we can only speculate that the disinfection properties of the surfaces might depend on the temperature difference of the surface and the dew point (Fig 3D, grey bars), where for maximum effectiveness of the photocatalytic effect the difference should be about 3 °C (Fig 3D, black dashed line). This is not supprising, since fogs of all types start forming when the air temperature and dewpoint of the air become nearly identical. This occurs through cooling of the air near the cool surface to a little beyond its dewpoint and the precipitation of water droplet from the air seldom forms when the dewpoint spread is about 3 °C. Other microclimatic parameters (S1 Fig) didn’t follow the relative reduction of *L*. *innocua*. This results shows that photocatalytic disinfection of surfaces can be made efficient in humid places of the food processing plant. Significant reduction of the number of bacteria was found in the number of remaining bacteria on nanotube coated surfaces versus uncoated control surfaces (Fig 3E) when we averaged reductions of bacteria across all the places in the food processing plant. The average number of bacteria on the surfaces with the nanotube coating was reduced more (Log_10_ = 2.92) than on the control surfaces (Log_10_ = 3.90), which can be expressed as relative percent reduction of %R = 80%. This is similar to the antibacterial activity of copper doped TiO_2_ coatings on *Methicillin Resistant Staphylococcus aureus* (MRSA) (Log_10_ = 4.2 under visible light irradiation and Log_10_ = 1.8 in darkness) owing to the generation of reactive oxygen species which should be a major factor which substantially reduces bacterial growth on glass surfaces[48]. However the demonstrated antibacterial activity was also high even in the dark, probably due to presence of copper particles alone. Similarly Xiaojin et al.[49] developed an innovative coating system based on polymers containing fine particles Cu and TiO_2_ nanoparticles. Coatings were applied to various substrates, including plastics, glass, and steel. During investigation antimicrobial properties and microhardness of coatings were studied. Coatings showed an effective antibacterial activity (reduction of *E*. *coli* by 10^6^ in 2 hours), where Cu and TiO_2_ particles contributed to the strengthening of the resistance, persistence and microhardness of surfaces to which they were applied.

For further quantification of the results we calculated the ratios of bacteria from the coated surfaces versus the control surfaces for all the measurements (Fig 3F, white bars). In such presentation of the results the antibacterial effect is reflected in the ratio to of less than 1. The distributions of survival ratios (Fig 3F, white bars) were clearly not normal. Since biological mechanisms often induce lognormal distributions [50], for example when exponential growth is combined with further symmetrical variation such as initial concentration of bacteria [51–54], we fit our data with a log normal distribution (Fig 3F, dashed line). The lognormal fits of the histograms fit best the survival ratios also when compared to other distributions.

### Antimicrobial activity in presence of repeated daily contamination and washing

**Fig 4:**
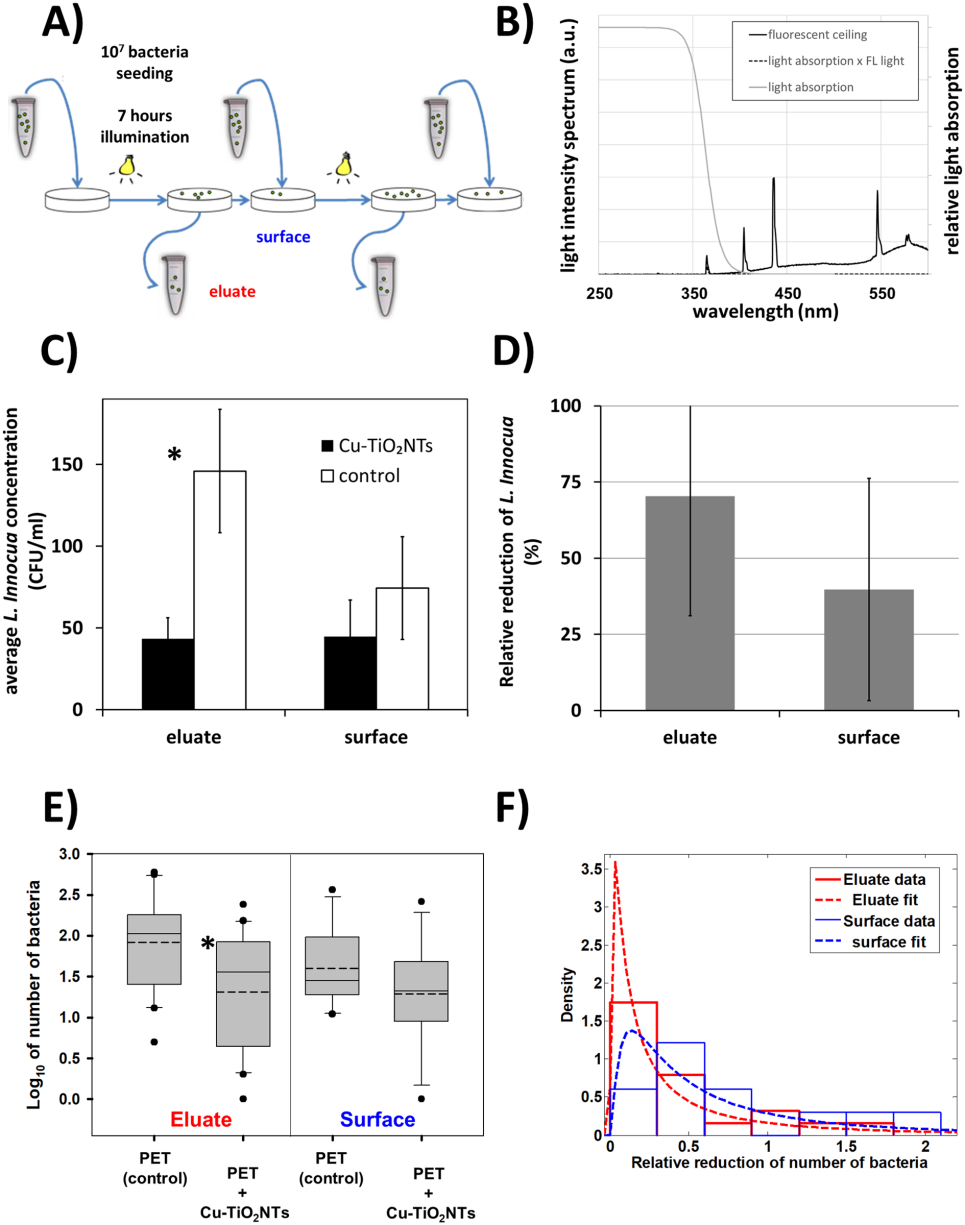
Survival of Listeria innocua under repeated daily contamination and washing. Reduction of number of bacteria Listeria innocua was measured on a polyethylene terephthalate (PET) surface with antibacterial nano coating (PET+Cu-TiO_2_NTs) or without the coating (PET) tested in a laboratory mimicking conditions in a food processing plant. **A)** Experimental setup scheme—Listeria innocua culture was continuously applied on surfaces as indicated by blue arrows pointing to a surface; **B)** spectrum of emited light from ceiling mounted fluorescent lights (black line); relative light absorption by the Cu-TiO_2_NTs (grey line); spectrum of absorbed light by the nanotubes (dashed line). **C)** Number of Listeria innocua shown on a logarithmic scale. On each surface 10^7^ bacteria were placed on PET slides. After 7 hours of exposure to ceiling mounted fluorescent lights remaining bacteria were transferred from the surfaces and colony forming units (CFU) were counted. Number of CFU on control surfaces is shown with white bars (PET control); Number of CFU on the nanoparticle coated surfaces is shown with black bars (PET + Cu-TiO_2_NTs); **D)** Relative reduction (%R) of bacteria as a consequnce of disinfecting action of nanoparticle coated surface, illuminated with ceiling mounted fluorescent lights (grey bars), calculated according to the Eq 1 in Materials and Methods section. **E)** Number of Listeria innocua in eluate from either control surface without nano coating (PET) or from a surface coated with copper doped TiO_2_ nanotubes (PET+Cu-TiO_2_NTs) presented in a boxplot, where the line in a box marks the median number of bacteria, while the dashed line marks the mean. The boundary of the box closest to zero indicates 25^th^ percentile, and the boundary farthest from zero indicates the 75^th^ percentile. Whiskers (error bars) above and below the box indicate the 90^th^ and 10^th^ percentiles, respectively. The outliers are shown as dots. Note the LOG scale. Reduction in number of bacteria in eluate for 0.6 orders of magnitude was statistically significant* (t = 3.018, with 38 degrees of freedom, two-tailed P-value = 0.00453, power of performed two-tailed test with alpha = 0.050 : 0.837). The reduction of bacteria on a surface was smaller than in eluate (t = 1.338, with 20 degrees of freedom, two-tailed P-value = 0.196, power of performed two-tailed test with alpha = 0.050 : 0.247). **F)** Histograms of the distribution (probability density function—PDF) of survival ratios in eluate (red) or on a surface (blue). Survival ratio was calculated as a ratio between the number of Listeria innocua from the surface with antibacterial coating (PET+Cu-TiO_2_NTs) and the number of Listeria innocua from a control surface without the coating (PET). For inefficient antibacterial coating survival ratio of 1 is expected. Dotted line is lognormal fit of the distributions with a maximum of the PDF at 0.02 and 0.13 for the reduction of the number of bacteria in eluate and on the surface, respectively.

Next we repeatedly inoculated and washed PET surfaces with *Listeria innocua* daily in laboratory conditions, in order to mimic daily contamination in food processing industry or surfaces in a household, as shown schematically in Fig 4A. After application, we left the bacteria on the surface for 7 hours while being exposed to low intensity light from fluorescent lamps on the ceiling (t = 7 h, j = 2.5 W/m^2^, A = 8 J (total light), A_<380nm_ = 80 mJ). As it can be seen from the measured light intensity spectrum of the fluorescent lamp (Fig 4B), the intensity of light with wavelengths below 380 nm (80 mJ) is only 1 percent of the total light intensity, the corresponding energy of the illumination of the nanotubes, which can induce the photocatalytic process of hydroxyl radical production, is therefore around 80 mJ in our experimental setup.

Although the antimicrobial effect was not as pronounced as in the food processing plant, average number of CFU/mL in eluate from the control surface was 146 ± 38, which is significantly higher than the number of CFU/mL in the eluate from the TiO_2_ nanotubes coated surface, which decreased to 43 ± 13 (Fig 4C, eluate) (t = 3.018, with 38 degrees of freedom, two-tailed P-value = 0.00453, power of performed two-tailed test with alpha = 0.050 : 0.837). In the 28 days lasting experiment the average number of CFU remaining on the control surface was 74 ± 31 CFU/mL, whereas the average number remaining on the nanotube coated surface was 45 ± 22 CFU/mL, which was not statistically different (Fig 4C, surface). The relative reduction of the number of *Listeria Innocua* in the eluate was 70% ± 39 in seven hours of exposure to the fluorescent lights, compared to a control surface (Fig 4D, eluate), whereas the reduction of bacteria remainig on a surface was negligible (Fig 4D, surface, see error bar extending to zero). All the data, from which averages were calculated, are shown in in the supplement (S1 Table) and in the Fig 4E in a form of a box plot, from which can be easily seen that the distribution of measurements is not normal (the mean and median are not overlaping). For more detailed quantification of the results we therefore calculated also the ratios of bacteria from the coated surfaces versus the control surfaces for all measurements (Fig 4F). The effect of the antibacterial coating on the survival of *Listeria innocua* is indicated by the ratio of less than 1. The distributions of survival ratios were also not normal as for the data in Fig 3F. We again fit the histograms of the survival ratios in the eluate and on the surface. The best fits of the data to the lognormal distribution indicate that the maximum of the probability density function is around 0.1, thus confirming that antibacterial coating is inhibiting the growth of *Listeria innocua* on TiO_2_ nanotube coated surfaces.

To test whether a small addition of copper is solely responsible for the antibacterial properties of the nanotube coated surface, the above experiments were also performed in the dark (see the Supplement, S2 Fig). In the experiment in the dark the number of colony forming units of *Listeria innocua* on nanotube coated as well as on control surface was the same, indicating that the reduction of bacteria that we observed originates from photocatalytic process of the TiO_2_ nanotubes.

## Conclusion

To implement advantages of germicidal disinfection with the use of ultraviolet light as well as antibacterial properties of copper-containing surfaces, we used recently characterized Cu^2+^-doped TiO_2_ nanotubes and achieved a stable deposition on several materials, including on the surface of polyethylene terephthalate (PET), as the one of the synthetic polymers commonly used in food processing industry. More importantly, we showed that such coating has disinfecting effect, with the number of remaining microorganisms significantly decreased on the surface coated with Cu^2+^-doped TiO_2_ nanotubes as well as in eluate from the coated surface, when illuminated with common ceiling mounted fluorescent lights. The disinfection properties of the nanotube coated surfaces depend on the intensity of the light, which should include wavelengths at about 370 nm, as well as on the temperature difference of the surface and the dew point, where for maximum effectiveness of the photocatalytic effect the difference should be about 3 °C.

Our results show that one dimensional nanomaterials, such as TiO_2_ nanotubes, can be employed for disinfection of polymer surfaces in the food industry, using cost effective illumination with existing fluorescent lights or additional low power light emitting diodes. Future use of such surfaces with antibacterial nano-coating and resulting sterilizing effect holds promise for such materials to be used in different environments or in better control of critical control points (HACCP) in food production as well as an improved biosecurity during the food manufacturing process.

## Supporting information

S1 Fig Microclimatic conditions in a food processing plant at different locations regarding the type of food processing. **A)** Temperature of air, surfaces on to which PET slides were placed, and dew point temperature; **B)** Relative and absolute air humidity; **C)** Intensity of ceiling mounted fluorescent lights at usual operating conditions; **D)** Airflow at different places in the food processing plant.
(TIF)

S2 Fig Survival of *Listeria innocua* in the dark. Reduction of number of bacteria Listeria innocua was measured on a polyethylene terephtalate (PET) surface with antibacterial nano coating (Cu-TiO_2_NTs) or without the coating (PET) tested in the dark in order to avoid the photocatalytic effect. The difference in the mean values of the two groups (PET: N = 6, mean = 3.15; Cu-TiO_2_NTs: N = 4, mean 3.29) is not great enough to reject the possibility that the difference is due to random sampling variability. There is not a statistically significant difference between the groups (P = 0.644).
(TIF)

S3 Fig EDS spectrum of Cu^2+^-doped TiO_2_ nanotube (Cu-TiO_2_NTs) sample. (TIF)

S1 Table Individual colony forming units counts (CFUs) of *Listeria innocua* under exposure to ceiling mounted fluorescent lights in a food processing plant. (TIF)

S2 Table Individual colony forming units counts (CFUs) of *Listeria innocua* on PET slides (controls and Cu-TiO_2_NTs coated) under exposure to ceiling mounted fluorescent lights in a food processing plant placed at different places either horizontally or vertically. (TIF)

S3 Table Individual colony forming units counts (CFUs) of *Listeria innocua* under exposure to ceiling mounted fluorescent lights in laboratory conditions with repeated daily contamination and washing. (TIF)
