# Supplementary figures and images for: Photocatalytic disinfection of surfaces with copper doped Ti02 nanotube coatings illuminated by ceiling mounted fluorescent light
Source: PLoS One. 2018 May 16;13(5):e0197308. doi: 10.1371/journal.pone.0197308 (PMC5955584; doi:10.1371/journal.pone.0197308)

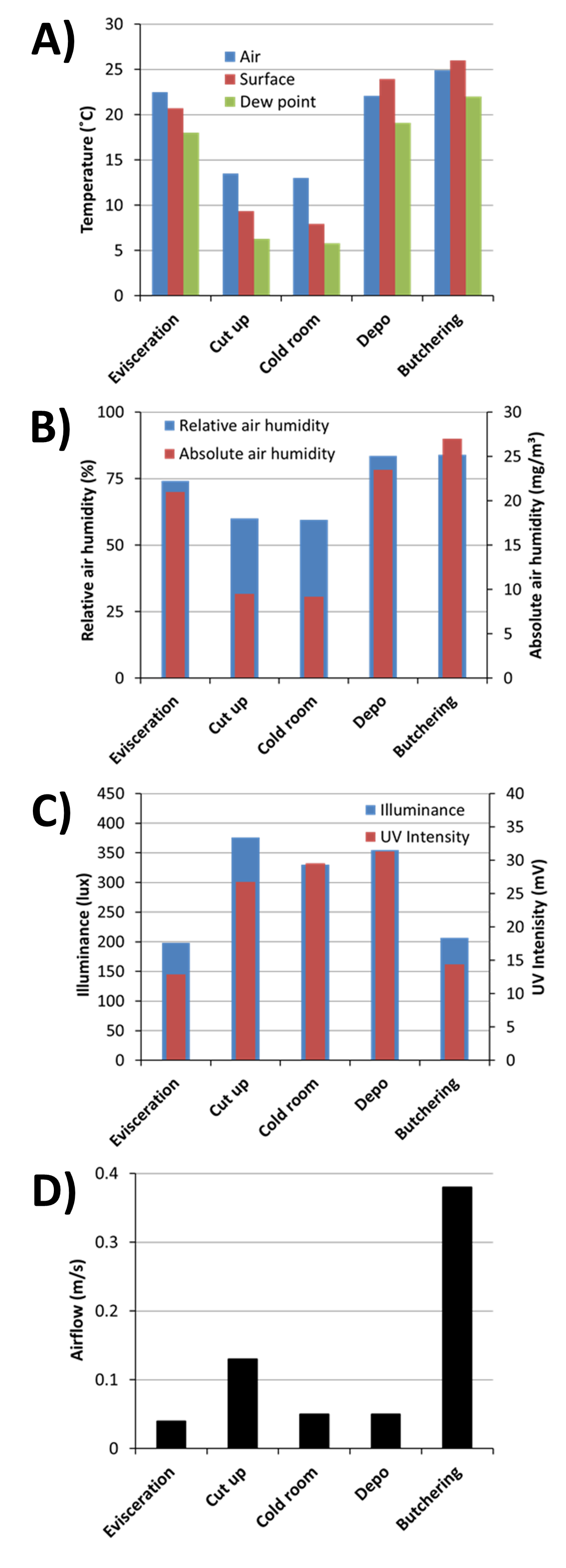

Supplement: S1 Fig — A) Temperature of air, surfaces on to which PET slides were placed, and dew point temperature; B) Relative and absolute air humidity; C) Intensity of ceiling mounted fluorescent lights at usual operating conditions; D) Airflow at different places in the food processing plant. (TIF) [file pone.0197308.s001.tif]

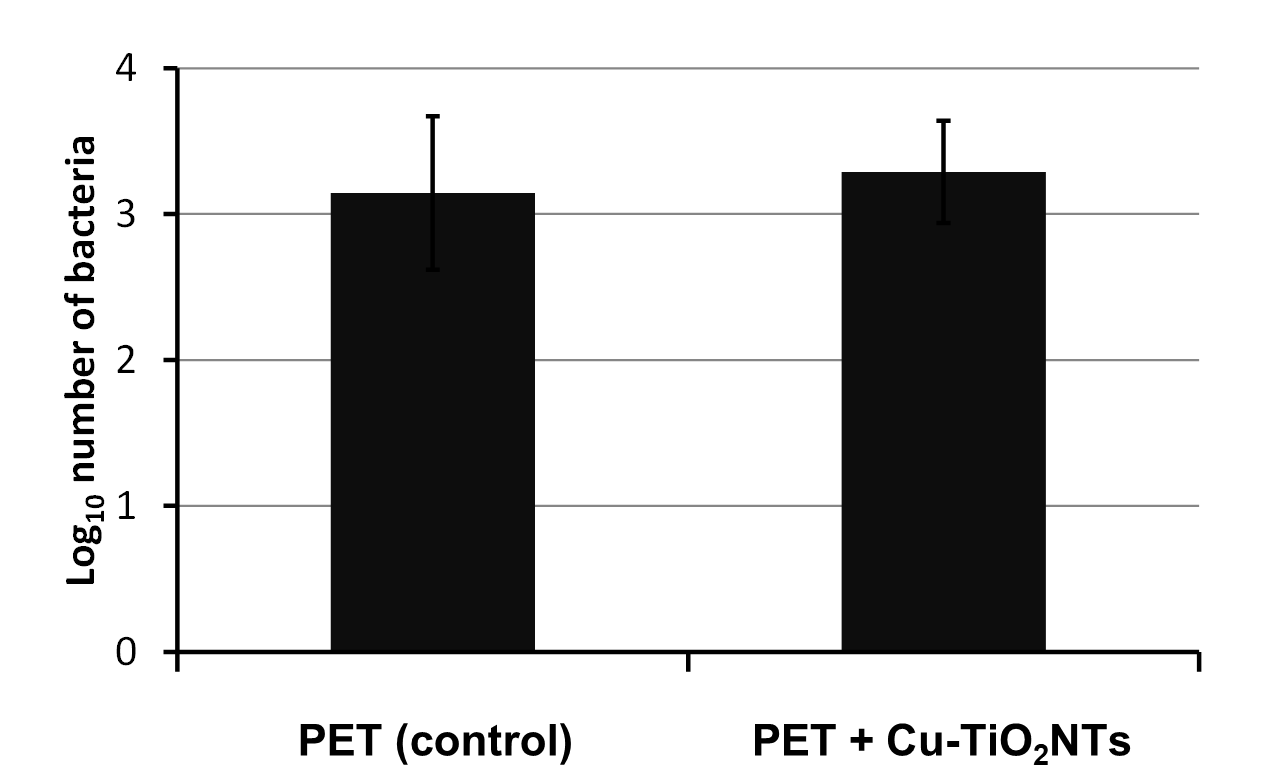

Supplement: S2 Fig — Reduction of number of bacteria Listeria innocua was measured on a polyethylene terephtalate (PET) surface with antibacterial nano coating (Cu-TiO2NTs) or without the coating (PET) tested in the dark in order to avoid the photocatalytic effect. The difference in the mean values of the two groups (PET: N = 6, mean = 3.15; Cu-TiO2NTs: N = 4, mean 3.29) is not great enough to reject the possibility that the difference is due to random sampling variability. There is not a statistically significant difference between the groups (P = 0.644). (TIF) [file pone.0197308.s002.tif]

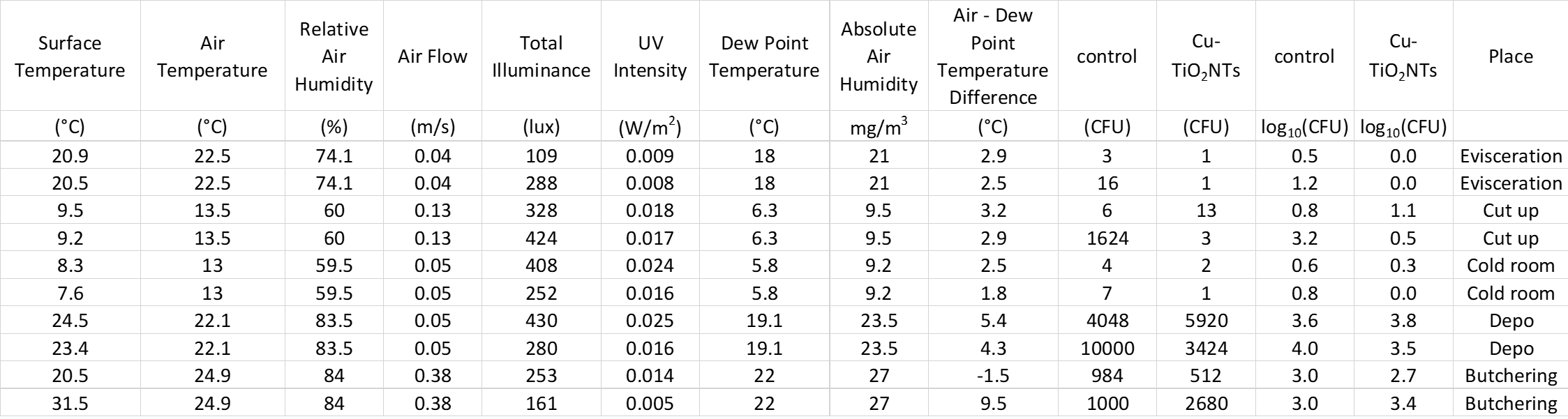

Supplement: S1 Table — (TIF) [file pone.0197308.s004.tif]

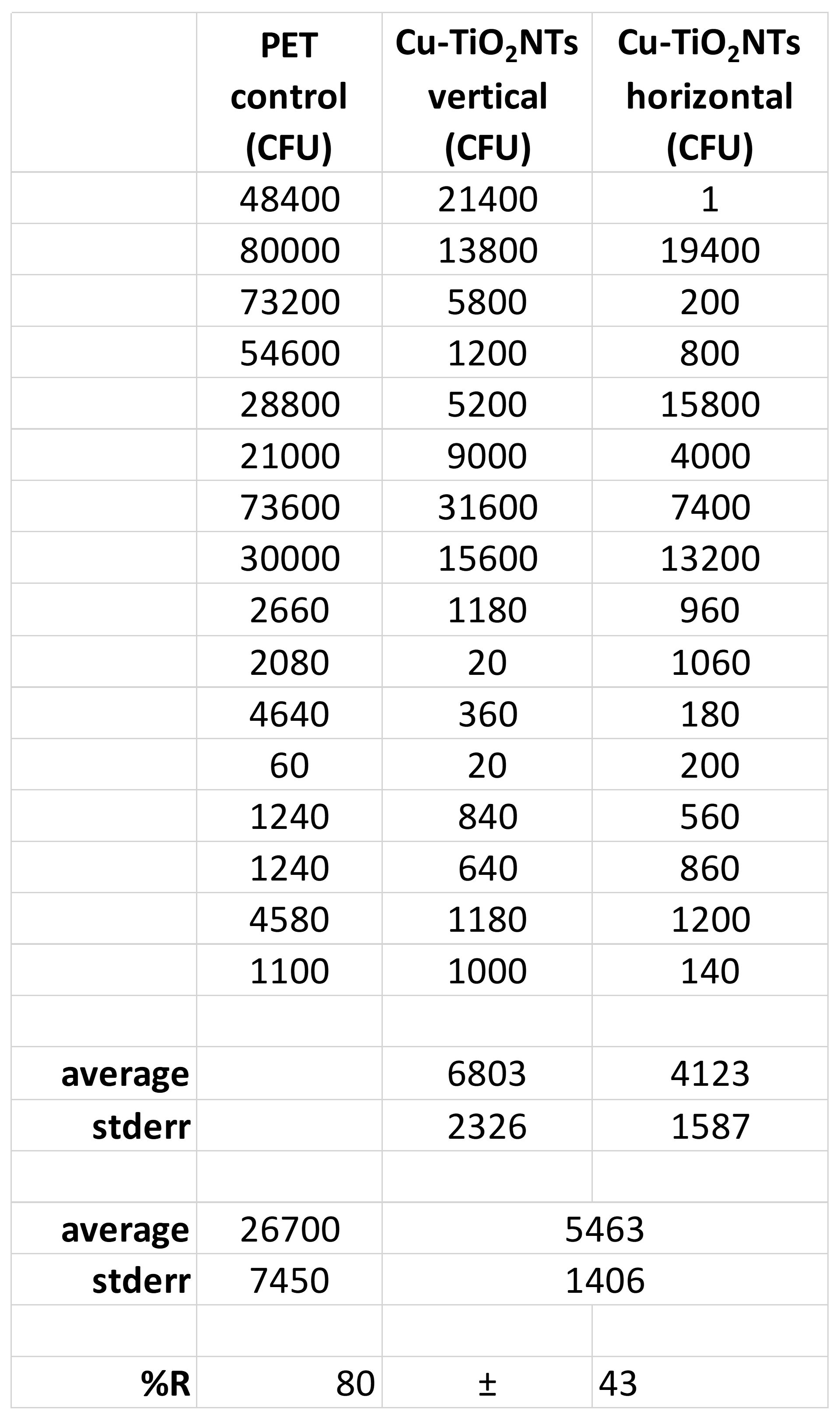

Supplement: S2 Table — (TIF) [file pone.0197308.s005.tif]

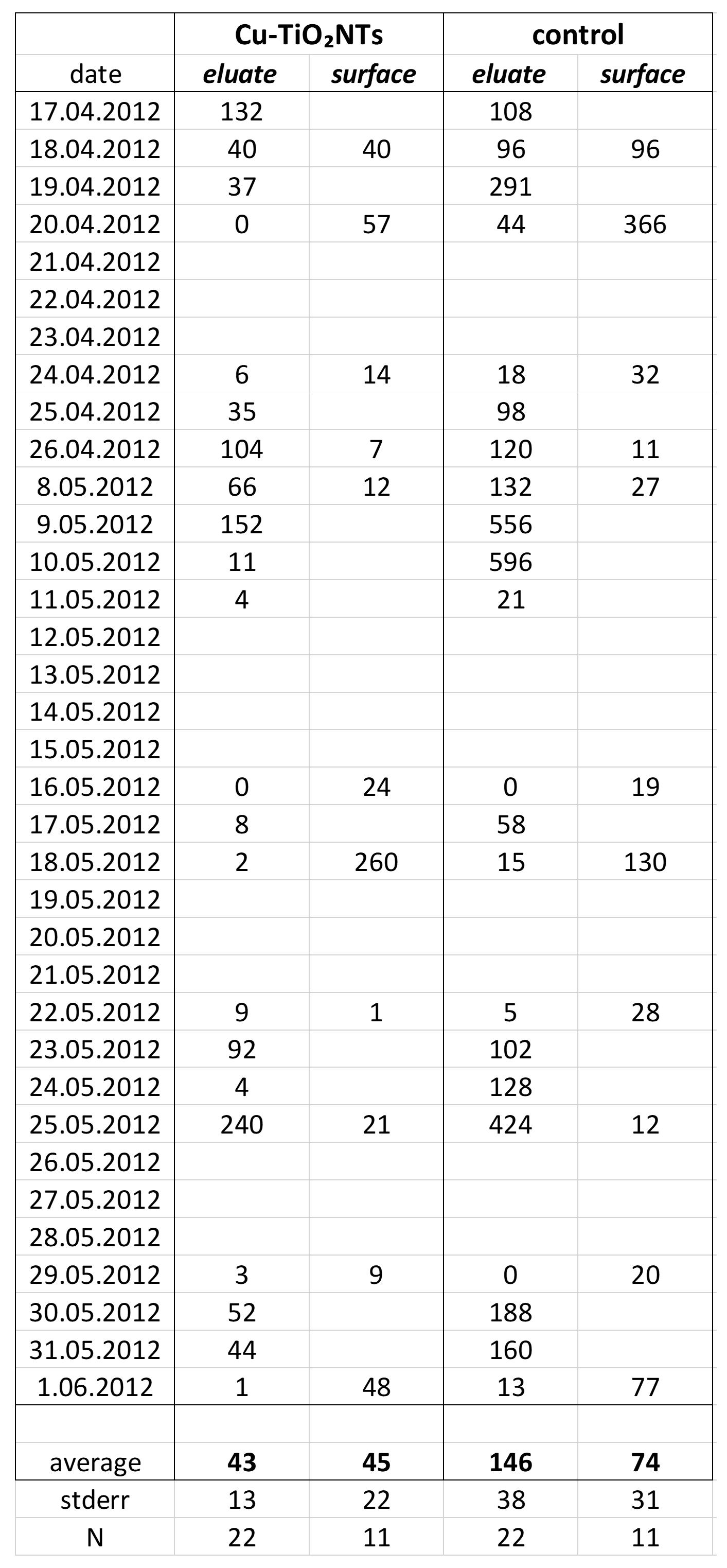

Supplement: S3 Table — (TIF) [file pone.0197308.s006.tif]
